# IMPACT OF THE BE EPIC: A PERSON-CENTERED COMMUNICATION INTERVENTION FOR HOME CARE WORKERS

**DOI:** 10.1093/geroni/igz038.2843

**Published:** 2019-11-08

**Authors:** Marie Y Savundranayagam, Kristine N Williams, Shalane Basque, J B Orange, Marita Kloseck, Vicki Schmall, Karen Johnson

**Affiliations:** 1 Western University, London, Ontario, Canada; 2 University of Kansas Medical Center, Kansas City, Kansas, United States; 3 Aging Concerns, West Linn, Oregon, United States; 4 McCormick Dementia Services, London, Ontario, Canada

## Abstract

The current study assessed the impact of Be EPIC, an innovative, evidence-informed and theoretically-grounded 6-week person-centered communication intervention for personal support workers (PSWs) caring for persons with dementia. Be EPIC focuses on [E]nvironment contexts for using [P]erson-centered communication, while considering client relationships ([I] matter too), and [C]lients’ abilities, life history and preferences during routine care. A pre- post-Be EPIC comparative design included an intervention (n=13) and a 6-week waitlist control group (n=10) who completed the same communication-related questionnaire. A Two-Way Mixed ANOVA showed a significant group by time interaction for perceived communication skill (F(1, 21) = 4.67, p = .042, ηp2= .18). Simple main effects analysis showed that participants who completed Be EPIC reported feeling more confident in communicating with persons with dementia (Mpre = 13.46; SD = .76; Mpost = 16.31, SD = .85). There was no significant change in the control group. Similarly, there was a significant group by time interaction for perceived helpfulness of communication strategies (F(1, 21) = 6.23, p = .021, ηp2 = .23). Simple main effects analysis showed that participants who completed Be EPIC reported significant increases in the helpfulness of effective communication strategies (Mpre = 36.92; SD = 3.42; Mpost = 43.15, SD = 3.21), with no significant change among controls. Findings indicate that Be EPIC enhanced PSWs’ confidence in communicating with persons with dementia and enhanced their perception of the helpfulness of effective communication strategies.

